# Multi-tissue transcriptional changes and core circadian clock disruption following intensive care

**DOI:** 10.3389/fphys.2022.942704

**Published:** 2022-08-15

**Authors:** Henry C. Hollis, Julian N. Francis, Ron C. Anafi

**Affiliations:** ^1^ School of Biomedical Engineering and Health Systems, Drexel University, Philadelphia, PA, United States; ^2^ Department of Mathematics, Howard University, Washington, DC, United States; ^3^ Division of Sleep Medicine and Chronobiology and Sleep Institute, University of Pennsylvania, Philadelphia, PA, United States

**Keywords:** circadian rhythm, circadian disruption, transcription, gene correlation analysis, critical care, critical illness, ICU, intensive care unit

## Abstract

**Objective:** Both critical illness and current care have been hypothesized to upset daily rhythms and impair molecular circadian function. However, the influence of critical illness on clock function in different tissues and on circadian output genes are unknown. Here we evaluate the effect of critical care and illness on transcription, focusing on the functional organization of the core circadian oscillator.

**Methods:** We downloaded RNAseq count data from the Genotype-Tissue Expression (GTEx) project. Treating mechanical ventilation as a marker for intensive care, we stratified samples into acute death (AD) and intensive care (IC) groups based on the documented Hardy Death Scale. We restricted our analysis to the 25 tissues with >50 samples in each group. Using the edgeR package and controlling for collection center, gender, and age, we identified transcripts differentially expressed between the AD and IC groups. Overrepresentation and enrichment methods were used to identify gene sets modulated by intensive care across tissues. For each tissue, we then calculated the delta clock correlation distance (ΔCCD), a comparative measure of the functional organization of the core circadian oscillator, in the both the AD and IC groups. The statistical significance of the ΔCCD was assessed by permutation, modifying a pre-existing R package to control for confounding variables.

**Results:** Intensive care, as marked by ventilation, significantly modulated the expression of thousands of genes. Transcripts that were modulated in ≥75% of tissues were enriched for genes involved in mitochondrial energetics, cellular stress, metabolism, and notably circadian regulation. Transcripts that were more markedly affected, in ≥10 tissues, were enriched for inflammation, complement and immune pathways. Oscillator organization, as assessed by ΔCCD, was significantly reduced in the intensive care group in 11/25 tissues.

**Conclusion:** Our findings support the hypothesis that patients in intensive care have impaired molecular circadian rhythms. Tissues involved in metabolism and energetics demonstrated the most marked changes in oscillator organization. In adipose tissue, there was a significant overlap between transcripts previously established to be modulated by sleep deprivation and fasting with those modulated by critical care. This work suggests that intensive care protocols that restore sleep/wake and nutritional rhythms may be of benefit.

## Introduction

Daily or circadian cycles in biology modulate much of physiology and behavior (Takahashi et al., 2008). Driven by endogenous biological clocks, circadian rhythms coordinate internal cellular processes, both with the external environment and with each other. In both mice (Zhang et al., 2014) and humans (Ruben et al., 2018), nearly 50% of protein encoding transcripts show daily rhythms in one or more tissues. Moreover, tissue specific circadian output genes are highly enriched for disease associated genes and drug targets. Circadian modulation of metabolism and immune function appear particularly robust (Zhang et al., 2016; Haspel et al., 2020).

In almost every human cell, molecular circadian rhythms are generated by a transcriptional translational feedback loop (TTFL). (Takahashi et al., 2008). Transcriptional activators CLOCK and ARNTL bind the promoters of EBOX containing targets including the Period (PER1, PER2, and PER3) and Cryptochrome (CRY1 and CRY2) genes. After protein translation and a series of post-translational modifications, the PER and CRY proteins are actively translocated, return to the nucleus, and ultimately inhibit their own translation by the CLOCK:ARNTL complex (Kume et al., 1999). Over time CRY and PER protein degradation removes their repressive influence, allowing the cycle to begin anew the next day. This simple delayed feedback loop has multiple levels of control (Takahashi, 2016): The stability of the CRY proteins is modulated by the opposing action of two ubiquitin ligases: F-Box and leucine rich repeat proteins 3 and 21 (FBXL3 and FBXL21) (Hirano et al., 2013; Yoo et al., 2013). Phosphorylation by kinases such as casein kinase 1 epsilon (CK1ε), casein kinase 1 delta (CK1δ) and cyclin-dependent kinase 5 (CDK5), orchestrate PER protein turnover (Lowrey et al., 2000) along with interactions between PER and CRY and their translocation back to the nucleus (Brenna et al., 2019). Additional regulation is provided by interacting feedback loops (Preitner et al., 2002; Bugge et al., 2012), allosteric protein-protein interactions (Cal-Kayitmazbatir et al., 2020), and epigenetic modifications. (Etchegaray et al., 2003; Nakahata et al., 2008; Katada and Sassone-Corsi, 2010; Koike et al., 2012; Greco et al., 2020; Li et al., 2020). Notably these additional levels of control have been hypothesized to link circadian rhythms with cellular metabolic state (Aguilar-Arnal and Sassone-Corsi, 2013; Milev and Reddy, 2015; Patke et al., 2019).

The utility of these clocks likely depends on the ability to entrain them to the external environment and with each other. The “master” clock in the supra chiasmatic nuclei of the hypothalamus receives photic information and helps coordinate peripheral clocks through a combination of autonomic and hormonal signals (Bass, 2012). Other tissues are more easily entrained by different time giving signals or zeitgebers. While the SCN, for example, is closely entrained to the light-dark cycle, clocks in the liver and even skin are more tightly entrained to feeding rhythms (Vollmers et al., 2009; Wang et al., 2017). The sleep/wake cycle, perhaps the most outwardly obvious of all circadian regulated behaviors, itself contributes substantially to daily rhythms in transcription (Möller-Levet et al., 2013; Archer et al., 2014) and may synchronize peripheral clocks through autonomic and temperature signals (Anafi et al., 2013; Hoekstra et al., 2019).

Patients with need of intensive care are, almost by definition, subject to a host of severe inflammatory and metabolic stressors. The decision to admit patients to intensive care is based on a need for aggressive interventions and/or continuous monitoring. These patients are typically at risk for hypoxia or decreased systemic perfusion, often related to trauma, acute neurologic events, or profound illness with systemic inflammatory response (Smith and Nielsen, 1999). Indeed, critical care and illness have been found to influence tissue physiology to an extent that gene expression can predict subjects’ pre-mortem critical care status (Somekh et al., 2022). Disease related disruption of the hypothalamic-pituitary-stress axis, along with associated changes in body temperature, autonomic tone, and hormone secretion are well established, and all have been implicated in peripheral circadian entrainment (Nicolaides et al., 2014, 2017; Astiz et al., 2019). Moreover, vasopressors, steroids, sedatives, and other treatments often used in intensive care may directly modulate these entraining signals (Brainard et al., 2015; Astiz et al., 2019; Maas et al., 2020b; Haspel et al., 2021). Of equal importance, contemporary intensive care medicine takes place in an environment often devoid of normal time-entraining cues with poor lighting, absent or irregular enteric nutrition, constant sedation, and/or sleep disruption (Freedman et al., 2001; Gabor et al., 2003; Brainard et al., 2015). Thus, several groups have hypothesized that patients in intensive care are likely to have disrupted molecular clocks and this disruption may, in theory, compound the existing immune and metabolic insults (Telias and Wilcox, 2019; Jobanputra et al., 2020; Haspel et al., 2021). Initial studies have demonstrated disruption of sleep/wake and core-body temperature rhythms (Gazendam et al., 2013) among IC patients and absent or altered circadian patterns of serum melatonin and cortisol (Mundigler et al., 2002; Kiss et al., 2015; Maas et al., 2020b). Similar results have been obtained when comparing the temporal patterns in whole blood gene expression among IC patient cohorts and controls, both at the level of select core clock genes (Diaz et al., 2020) and the larger transcriptome (Maas et al., 2020a). Indeed, agglomerating data from multiple patients to create a composite temporal profile, Diaz et al. found that blood born rhythms in *CLOCK*, *ARNTL*, and *PER2* detectable upon admission were lost after 1 week in the ICU.

The influence of critical illness and critical care on circadian and transcriptional regulation in other tissues, however, remains unclear. Of course, practical safety limitations prohibit repeated sampling of internal organs in ill (or healthy) patients. However, recently several tools have been developed to characterize rhythms and circadian function in agglomerated non-time stamped data.

## Materials and methods

RNAseq count data was downloaded from the Genotype-Tissue Expression project (GTEx Analysis V8) (Aguet et al., 2020). Using Hardy Death Scale scores documented in the GTEx data, we divided subjects into two groups, separating subject who died in an acute manner from those who died on a ventilator. Of the subjects ventilated, the median ventilation time was 76.39 h (median absolute deviation ± 32.4 h). A Hardy Death score of one describes patients who died in a violent or sudden manner due to accident or trauma, while a score of two describes patients who were reasonably healthy until a sudden terminal medical event (i.e., cardiac arrest). Subjects with scores of one or two are estimated to have a terminal phase <1 h and these subjects were combined to constitute the acute death (AD) group. Subjects with a Hardy Death score of zero died on a ventilator and constitute the Intensive Care (IC) group. Subjects who did not fall into the above-mentioned Hardy Death Scale categories were discarded for this analysis (categories three and four). While the GTEx dataset includes data from multiple tissues from each subject, different tissues were sampled from each subject. As a result, some tissues had very few samples from the AD or IC groups. As our analysis is focused on assessing difference between the IC and AD groups, we restricted our analysis to the 25 tissues with greater than 50 samples in each (IC and AD) group.

The edgeR package in R (R version 4.1.0) was used to assess differential expression between the AD and IC groups. Read counts were normalized prior to eliminating data from subjects with Hardy scale three and four deaths. Lowly or non-expressed genes were filtered out as detailed in the edgeR documentation (Robinson et al., 2010; Chen et al., 2016). The design matrix for differential expression analysis included collection center, age, sex and manner of death (IC vs. AD). The age assigned to each subject was the mean of the 10-years age range provided by GTEx. Expression values for each transcript were fit to a negative binomial distribution with a generalized log-linear model as per the edgeR user guide (Chen et al., 2022). Differentially expressed genes were found by performing a quasi-likelihood F test (edgeR: glmQLFTest). Two distinct criteria for differential expression were invoked: The more strict criteria required both a statistically significant difference in expression between the AD and IC groups (Benjaminni Hochberg q value BH. q < 0.050) (Benjamini and Hochberg, 1995) and a marked absolute change in expression of >50%. The more lenient criteria required a statistically significant difference in expression between the AD and IC groups (BH.q < 0.05) but did not require a specific fold change cutoff. Two different gene sets were thus identified for downstream analysis: The first list included all transcripts that met the strict criteria for differential expression in ten or more tissues. The second list included all genes that met the more lenient criteria but in a much larger number of tissues (75%, or 19 or more tissues).

The EnrichR R package was used to perform overrepresentation analysis and identify annotated gene sets overrepresented among transcripts differentially expressed in the IC group (Chen et al., 2013; Kuleshov et al., 2016). Annotated gene sets from the Gene Ontology (GO) GO_Biological_Process_2021 and KEGG_2021_Human gene set libraries were analyzed.

Clock correlation distance (CCD) is a metric designed to measure the functional organization of the core circadian oscillator (Shilts et al., 2018). The CCD method compares a matrix of Spearman correlation coefficients between 12 core clock genes to a reference correlation matrix provided by Shilts et al. To compare oscillator organization between two groups, the CCD of the treatment group is subtracted from the CCD of the control group yielding a ΔCCD. The statistical significance of this metric is assessed by bootstrap, shuffling the condition labels and recording the number of instances a greater or equal ΔCCD is observed (Supplementary Figure S1). A measure of significance is obtained using the method of Phipson and Smyth (2010).

We calculated the ΔCCD between acute (AD) and intensive care (IC) patients for each tissue. However, the ΔCCD metric can also be influenced by confounding variables. To account for confounding due to different collection center distributions in the AD and IC groups we modified the bootstrap procedure used to assess statistical significance. The modified test shuffles the condition labels (AD vs. IC) among subjects from each collection center independently. It then joins the permutations created for each collection center to create a combined permutation. This process maintains the same collection center distribution in each of the permutations (Supplementary Figure S2) used for comparison. This process thus directly accounts for the role of collection center in assessing the significance of any observed difference in the ΔCCD. We extended this procedure to account for the influence of both subject sex and collection center. One thousand bootstrap permutations were used to generate a null distribution simultaneously including both covariates. An alternative method to control for confounding variables was also performed using Combat-Seq in the sva R package (Leek et al., 2021). In this case, covariate normalized expression measures (adjusting for age range, gender, and collection center) were directly processed with the original ΔCCD method. An overview of the full informatic experimental design in shown in Supplementary Figure S3.

## Results

We identified 25 tissues with more than 50 samples in each of the AD and IC groups (Table 1). Among those 25 tissues, a mean of 421.2 samples were collected. These samples were collected from two primary GTEx collection centers. The median age of the subjects ranged from 50–62 depending on tissue. A summary of the data is given in Table 1.

**TABLE 1:** Summary statistics of included subjects. AD and IC groups refers to Acute Death and Intensive Care groups, respectively. Samples collected from centers B1 and C1. MAD refers to the median absolute deviation of a variable from the median.

Tissue	AD group	IC group	AD group	IC group	AD group	IC group	AD group	IC group
	n	n	% Female	% Female	Median age ± MAD (years)	Median age ± MAD (years)	%B1 center	%B1 center
Adipose - Subcutaneous	197	351	23.9	36.5	59 ± 6.0	50 ± 9.0	56.9	83.5
Adipose - Visceral	152	313	20.4	38	59 ± 6.0	50 ± 9.0	57.2	85
Adrenal Gland	52	195	28.8	40.5	59 ± 5.0	51 ± 8.0	51.9	93.8
Artery - Aorta	106	266	26.4	36.5	59 ± 7.0	52 ± 8.5	46.2	85
Artery - Coronary	54	157	27.8	43.3	59 ± 7.0	54 ± 7.0	42.6	87.9
Artery - Tibial	188	369	21.3	36.6	58 ± 6.5	50 ± 9.0	53.7	86.4
Breast	125	261	24	44.8	59 ± 6.0	50 ± 9.0	52.8	83.5
Cells - Cultured fibroblasts	139	277	25.2	38.6	58 ± 6.0	50 ± 9.0	45.3	80.5
Colon - Sigmoid	82	236	23.2	40.7	62 ± 5.0	50 ± 10.0	57.3	88.6
Colon - Transverse	51	316	23.5	38.3	59 ± 6.0	50 ± 9.0	52.9	87.3
Esophagus - Gast. Junc	89	253	20.2	37.9	56 ± 6.0	52 ± 8.0	49.4	87
Esophagus - Mucosa	116	366	27.6	35.8	59 ± 6.5	50 ± 9.0	53.4	84.7
Esophagus - Muscularis	98	363	19.4	38	59 ± 6.0	50 ± 9.0	51	87.1
Heart - Atrial Appendage	148	203	20.9	38.9	59 ± 6.0	54 ± 7.0	56.1	82.3
Heart - Left Ventricle	123	246	18.7	38.2	59 ± 6.0	54 ± 7.0	52	84.1
Liver	106	85	20.8	35.3	58 ± 6.0	52 ± 7.0	54.7	82.4
Lung	182	299	23.1	34.8	58 ± 6.0	52 ± 8.0	54.9	82.6
Muscle - Skeletal	234	423	21.8	37.1	59 ± 6.0	50 ± 9.0	58.1	84.4
Nerve - Tibial	185	327	23.2	36.4	59 ± 6.0	50 ± 10.0	53	83.2
Prostate	67	149	0	0	59 ± 7.0	50 ± 10.0	56.7	89.3
Skin - Not Sun Exposed	183	313	19.7	38.7	59 ± 6.0	50 ± 9.0	60.1	84.3
Skin - Sun Exposed	220	351	23.6	37.6	59 ± 6.0	51 ± 9.0	57.3	84
Testis	129	201	0	0	59 ± 6.0	50 ± 10.0	60.5	87.6
Thyroid	196	357	23	38.4	59 ± 6.0	50 ± 9.0	56.1	84.9
Whole Blood	217	412	24	38.3	59 ± 6.0	50 ± 10.0	58.5	86.4

**FIGURE 1:**
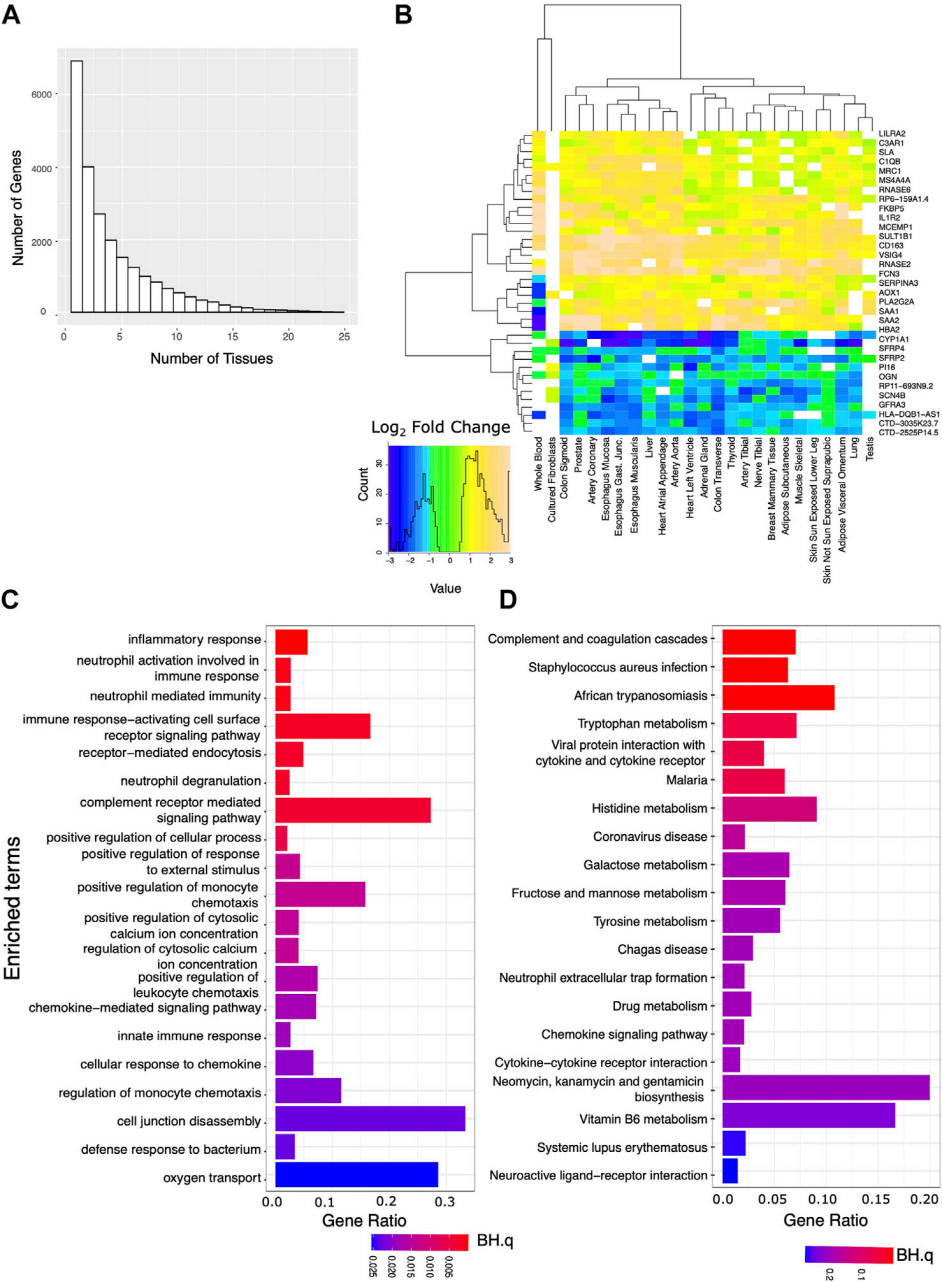
Analysis of genes strongly affected by critical care and illness. We analyzed the set of genes that were both significantly (BH.q < 0.05) and strongly (fold change of >1.5 or log_2_ fold change 0.585) differentially expressed between the IC and AD groups across tissues. **(A)** Histogram of the number of tissues for which any given gene meets these criteria. **(B)** Log_2_ fold changes of genes significantly differentially expressed between AD and IC groups (in 22 or more tissues) shown across tissue type. Transcripts appear to be consistently up or down regulated across tissue type. **(C)** GO BP and **(D)** KEGG pathways overrepresented in IC differentially expressed genes that met the above criteria in 10 or more tissues.

Using edgeR and a model that accounts for confounding categorical factors including sample collection center, subject sex, and age range, we analyzed the expression data for each tissue. We first identified transcripts that showed marked and significant changes in expression when comparing the AD and IC groups. Requiring strict statistical significance after adjusting for multiple testing (BH.q < 0.05) and an expression fold change of at least 50% between the two groups, we identified hundreds to thousands of differentially expressed genes in each tissue (Supplementary File S1). We then identified a set of these genes that were markedly differentially expressed in 10 or more tissues (Figure 1A). Notably these transcripts showed a consistent direction of expression change across tissues (Figure 1B). We used EnrichR to identify GO Biological Processes or KEGG annotated gene sets overrepresented among genes that markedly differed between the AD and IC group across ≥10 tissues. Consistent with the expectation that patients requiring critical care were under profound physiologic stress, pathways related to inflammation and complement activation along with metabolic pathways were overrepresented.

**FIGURE 2:**
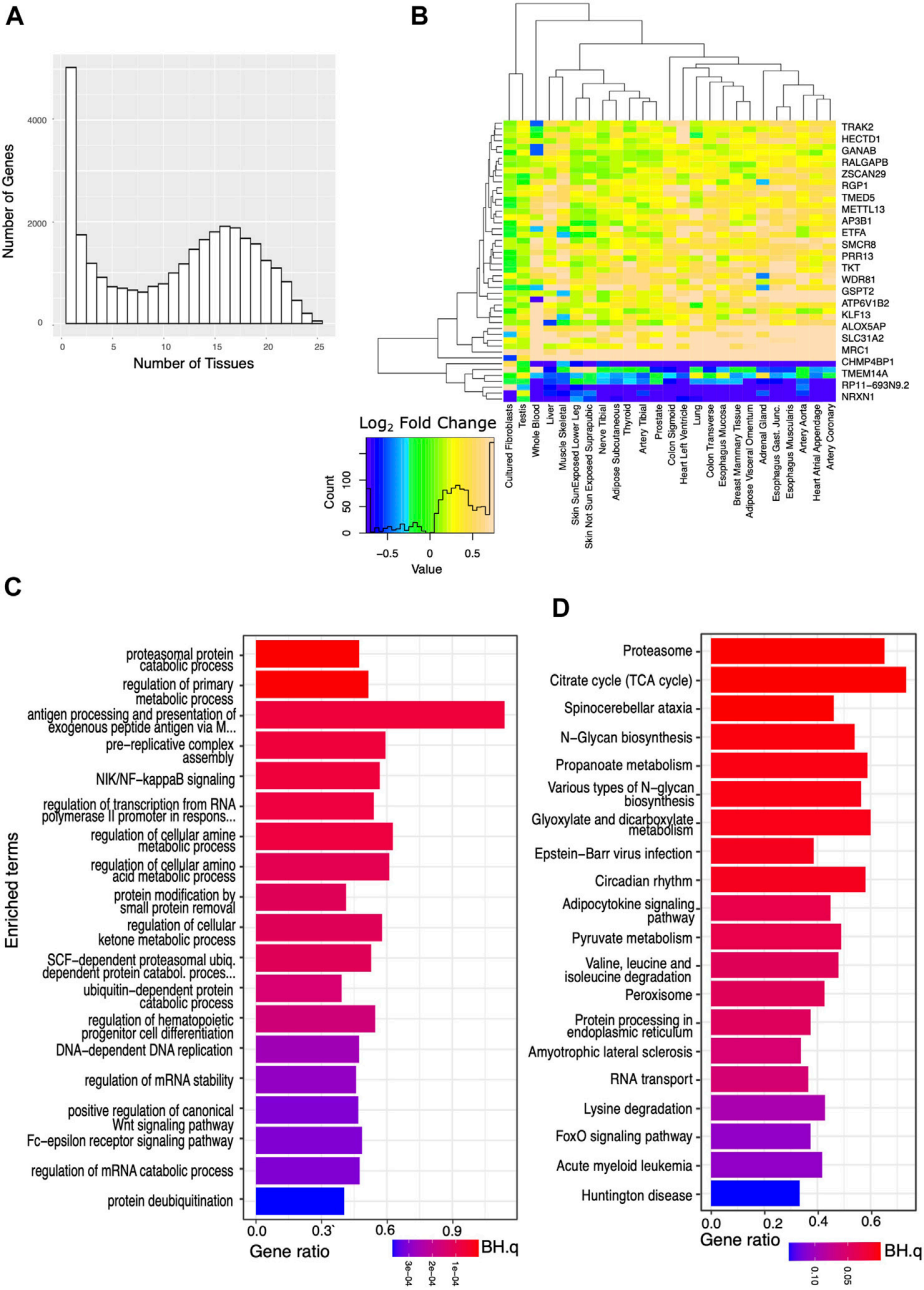
Analysis of genes significantly affected by critical care across a wide variety of tissues. We analyzed the set of genes that were significantly (BH.q < 0.05) differentially expressed in the IC and AD groups. **(A)** Histogram of the number of tissues for which any given gene meets these criteria. **(B)** Log_2_ fold changes of genes significantly differentially expressed between AD and IC groups (in 25 tissues) shown across tissue type. Transcripts appear to be consistently up or down regulated across tissue type. **(C)** GO BP pathways and **(D)** KEGG pathways enriched in IC differentially expressed genes that met the above criteria in ≥75% of tissues. Note appearance of circadian rhythm among the most significantly (*p* < 0.0003, BH. q < 0.01) enriched pathways.

Applying more lenient criteria and identifying transcripts with a significant difference in expression between the AD and the IC groups (BH.q < 0.05), but not requiring a specific fold change, we again identified thousands of transcripts in each tissue (Figures 2A,B). We then identified a set of transcripts that, although showing these more modest differences between the AD and IC groups, were affected in an even greater number (≥75%) of tissues. Overrepresented KEGG and GO BP pathways identified among these genes reflected widespread changes in cellular metabolism, protein, and nucleic acid processing, mitophagy, and autophagy. Notably the KEGG pathway (Figure 2D) for circadian rhythm regulation was among the most significantly overrepresented pathways (BH.Q < 0.05).

**FIGURE 3:**
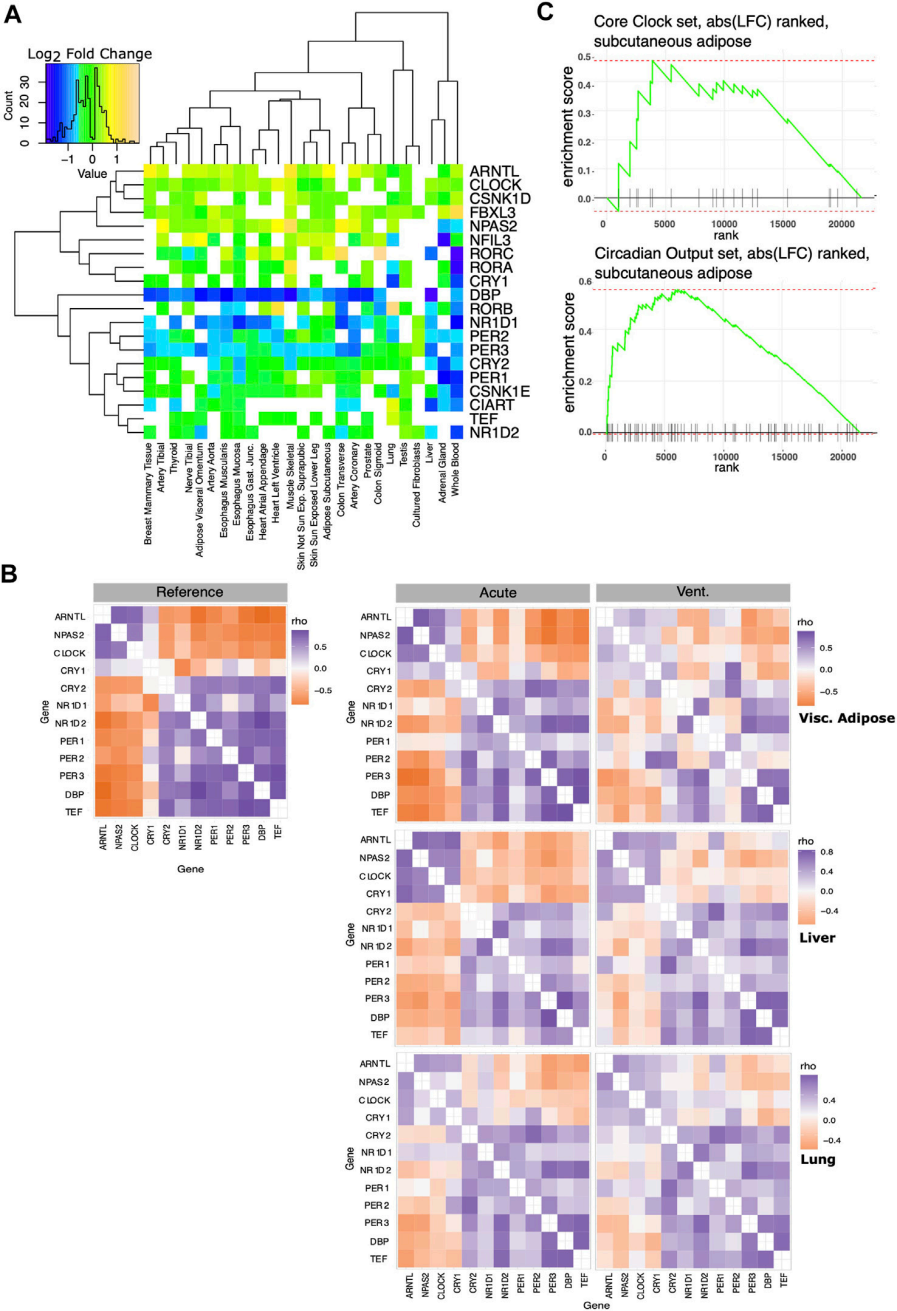
Effect of critical care on core clock organization and circadian output genes. A set of core clock genes was taken from the literature (Anafi et al., 2014; Shilts et al., 2018). **(A)** Log_2_ fold changes of core circadian clock genes significantly differentially expressed between AD and IC groups shown across tissues. Note that in most tissues, most core clock genes show a very small fold change. CCD matrices assess core clock organization and co-expression. **(B)** Left: the reference correlation matrix, used to calculate the CCD metric. Right: Example Spearman correlation matrices of core circadian clock genes in visceral adipose, liver and lung tissue. Clock function in visceral adipose is markedly decreased in the IC group, liver tissue shows a more moderate effect. ΔCCD analysis does not suggest a change in pulmonary oscillator organization following IC. **(C)** We ranked genes based on the log fold change between the AD and IC groups in subcutaneous adipose tissue. This was compared to lists of core clock and robust circadian output genes using fGSEA. Enrichment plot for core clock set (top), does not show significant enrichment. Enrichment plot for the robust circadian output genes shows significant enrichment (*p* < 0.01) as clock outputs were frequently ranked among the genes most strongly influenced by IC.

We defined a set of “core clock” genes combining the exemplar core clock components in (Anafi et al., 2014) with the clock genes used in (Shilts et al., 2018). In most tissues, the expression of genes in the core clock mechanism was modestly but significantly different in the IC vs. AD groups (Figure 3A). To help discern if the changes observed in circadian rhythm regulation reflect a significant change in oscillator function, we computed the ΔCCD, a comparative measure of the functional organization of the core circadian clock. The ΔCCD metric is based on a well conserved pattern of clock gene co-variance (Figure 3B Left). Tissues with a higher ΔCCD value display a greater disruption in the functional organization of the core oscillator when comparing the IC and AD groups. After pre-processing the expression data with Combat-Seq to adjust for age range, sex, and collection center, the ΔCCD suggested a significant decrease in oscillator function in the IC group in 14/25 tissues (Bonferroni corrected *p* ≤ 0.05). We then applied the more stringent, custom ΔCCD bootstrap procedure directly to the edgeR normalized count data. In 11/25 tissues, IC patients demonstrated disrupted core clock organization (Bonferroni corrected *p* ≤ 0.05). We did not have enough samples to adjust our liver analysis for gender. Thus, the liver analysis accounted only for collection center. Tissues with a higher ΔCCD showed a greater disruption in oscillator organization between IC and AD groups. Example CCD matrices showing clock organization in visceral adiposes, liver, and lung tissue–in both the IC and AD groups - are shown in Figure 3B (right). While the organization of the core clock in visceral adipose was markedly decreased in the IC group, liver provides an example of a tissue demonstrating a more moderate effect. Interestingly, while the IC group was defined by ventilator use, in the lung, core oscillator organization was relatively unchanged between the IC and AD groups. Regardless of the statistical method invoked, tissues associated with digestion and metabolism (Table 2) showed the largest decrease in clock function among IC patients.

**TABLE 2:** Influence of IC on clock correlation structure across tissues. Twelve core circadian clock genes have a well conserved signature correlation matrix in healthy mammalian tissue.

Tissue	ΔCCD (AD vs. IC)	ΔCCD Bonferroni corrected *p* value	Modified ΔCCD Bonferroni corrected *p* value
**Colon Transverse**	1.67	0.005	**0.050**
**Adipose Visceral**	1.66	0.005	**0.025**
**Esophagus Gast. Junc**	1.46	0.005	**0.025**
**Esophagus Mucosa**	1.35	0.005	**0.025**
**Colon Sigmoid**	1.28	0.005	**0.025**
**Esophagus Muscularis**	1.06	0.005	**0.025**
**Thyroid**	1.05	0.005	**0.025**
**Prostate**	1.03	0.110	^a^0.074
**Breast Mammary**	0.93	0.005	0.150
**Artery Coronary**	0.91	0.070	0.475
**Muscle Skeletal**	0.90	0.005	**0.025**
**Heart Atrial Appendage**	0.86	0.020	0.125
**Heart Left Ventricle**	0.85	0.025	**0.025**
**Liver**	0.83	0.290	^a^0.124
**Skin Not Sun Exposed**	0.75	0.005	**0.025**
**Lung**	0.71	0.230	0.200
**Adipose Subcutaneous**	0.71	0.030	**0.025**
**Artery Aorta**	0.68	0.055	0.400
**Artery Tibial**	0.62	0.025	0.075
**Nerve Tibial**	0.38	0.570	1
**Skin Sun Exposed**	0.32	0.405	1
**Testis**	0.31	0.730	^a^1
**Cells Cultured fibroblasts**	0.08	1	1
**Whole Blood**	0.04	0.715	1
**Adrenal Gland**	-0.90	1	1

a Modified ΔCCD *p*, value for liver are only corrected for center (as opposed to center and gender) given the limited number of samples.

CCD measures departure of a sample from that healthy correlation. All tissues had significant CCD values (Bonferroni p < 0.01) in the AD group (not shown). ΔCCD compares the difference in CCD values between two groups of samples (here AD and IC groups). Significance values are obtained through permutation. “ΔCCD p Value” represents significance from the unmodified procedure, using Combat to correct for covariates. Our modified and more stringent ΔCCD significance procedure directly accounts for gender and collection center covariates in the data.

Bonferoni-corrected *p* values <0.05 are bolded.

While the absolute expression changes in core clock genes were more modest, the altered correlation pattern captured by the ΔCCD suggest a functional change. In addition to defining a set of core clock genes, we defined a set of robust circadian output genes. Robust circadian outputs are human transcripts orthologous to transcripts that demonstrated statistically significant (JTK Cycle bHQ<0.05) circadian rhythms in ≥75% of tissues in the Mouse Circadian Atlas (Zhang et al., 2014). We then used Fischer’s exact test to see if either core clock or robust circadian output genes were overrepresented among those transcripts that were *markedly* (BH.q < 0.05 and >50% fold change) different between the IC and AD groups (Table 3). While core clock genes were not overrepresented among markedly IC affected genes in any tissue, 10 tissues showed an overrepresentation of robust clock output genes in IC affected genes (BH.q < 0.1)—consistent with altered oscillator function. In a related analysis we ranked the transcripts in each tissue by the log fold change observed between the IC and AD groups and used fGSEA (Korotkevich et al., 2021) to determine if core clock genes or robust circadian outputs were enriched among the transcripts most effected by critical illness (Figure 3C). The enrichment analysis, like the overrepresentation analysis, showed a stronger influence of IC on the expression of circadian outputs as compared to the absolute expression of core clock transcripts.

**TABLE 3:** Overexpression analysis of core clock and circadian output pathways in IC affected genes.

Tissue	Circadian output set BH.q values	Core clock set BH.q values
Skin Sun Exposed	**0.004**	1
Adipose Subcutaneous	**0.011**	1
Muscle Skeletal	**0.011**	1
Skin Not Sun Exposed	**0.011**	1
Artery Coronary	**0.047**	1
Liver	**0.047**	1
Testis	**0.047**	1
Artery Tibial	**0.08**	1
Breast Mammary Tissue	**0.08**	1
Nerve Tibial	**0.08**	1
Adipose Visceral	0.116	1
Thyroid	0.117	1
Lung	0.404	1
Artery Aorta	0.618	1
Esophagus Mucosa	0.618	1
Esophagus Muscularis	0.618	1
Colon Sigmoid	0.643	1
Heart Atrial Appendage	0.643	1
Adrenal Gland	0.657	1
Colon Transverse	0.657	1
Heart Left Ventricle	0.657	1
Whole Blood	0.657	1
Esophagus Gast. Junc	0.704	1
Cultured Fibroblasts	0.777	1
Prostate	1	1

For each tissue, Fisher’s exact test was performed to assess the overlap between the core clock pathway and genes significantly and highly modulated in the IC group for that tissue (BH.q < 0.05 and abs (LFC) > 0.58). The same approach was used to assess the overlap between a set of robust circadian outputs and the IC affected genes. The table shows the significance of the overlap in each tissue.

Benjamini-Hochberg *q* values <0.1 are bolded.

## Discussion

Multiple studies have shown than circadian rhythms in plasma cortisol, melatonin, and blood-based gene expression are disrupted among IC patients. Light, noise, steroids, vasopressors anesthetics, irregular nutrition, and benzodiazepines, have all been hypothesized to disrupt circadian rhythms, weaken entraining signals, or disrupt behaviors that may synchronize rhythms (Brainard et al., 2015; Maas et al., 2020b). However, prior work has generally been limited to blood and little is known about internal tissue physiology.

Here we show that thousands of genes are differentially expressed (BH.q < 0.05) across tissues in patients who died on a mechanical ventilation as compared to those who died suddenly. Consistent with the extreme physiologic stress of critical illness and critical care, the subset of genes modulated in ≥75% of tissues is enriched for pathways involving mitochondrial energetics, cellular stress, and immune function. Notably circadian regulation was also among the most enriched pathways. While the expression changes in core circadian clock genes observed between the IC and AD groups were relatively modest, we used the ΔCCD metric to assess the influence of these changes on core clock organization. The organization of core oscillator appeared to be significantly disrupted 11/25 tissues after using our modified ΔCCD procedure to correct for covariates. Among the most highly dysregulated tissues were those involved in digestion and metabolism, including various types of adipose, thyroid, esophageal, and colon tissue.

It is important, however, to note the limits of this analysis. The de-identified GTEx data available for public download does not provide time-course information. As a result, our study does not purport to describe the circadian transcriptome. Even if we were to obtain time-of-death information for each autopsy sample, the ability to agglomerate these data to re-construct a circadian transcriptome would require that the IC subjects are all well entrained to the shared external environment. Indeed, it is the assumption of shared entrainment that justifies using a 2:00 a.m. sample from one patient as follow up for a 1:00 a.m. sample from another. Yet this assumption is clearly in doubt in the IC cohort. Thus, unlike previous assessments of blood-based rhythms in IC patients, we did not attempt to assess the entrainment.

The core circadian clock is composed of set of activators (e.g. CLOCK and ARNTL) that induce the transcription of a set of repressors (e.g. *PER* and *CRY* family genes). This internal architecture imparts a well conserved temporal ordering among core clock genes with the activators sharing similar temporal expression patterns and being out of phase with repressors. As a result, the expression of activators tends to be positively correlated across samples and negatively correlated with repressors. For example, even combining data from night-shift and day-shift workers, we would still expect to find the expression of *ARNTL* anticorrelated with the expression of *PER2*. The CCD metric evaluates this correlation structure and the ΔCCD metric provides a prima-facie, comparative assessment of clock organization. Our ΔCCD analysis shows evidence of disrupted oscillator organization in IC patients across a wide variety of tissues. Moreover, we find that the homologues of clock outputs that showed strong circadian rhythms across a wide variety of mouse tissues were highly enriched among those transcripts that differed most strongly between the AD and IC groups. This provides additional, suggestive evidence that the change in core clock organization among IC patients has a functional consequence.

Great effort was made in controlling for age, sex, and collection center. Our modified ΔCCD bootstrap procedure is far more stringent as compared to the original ΔCCD procedure that used Combat corrected expression (Table 2). Combat adjusts the expression of individual genes into a “batch-less distribution.” However, it does not address the concern that the correlation between genes may itself be disturbed by a confounding variable. Moreover, it assumes that the temporal sampling of both batches is similar. Our more conservative approach attempts to address these concerns. Regardless of methodology, IC appears to have a clear influence on oscillator organization and a strong influence on clock output genes. Nevertheless, listed confounders may still contribute to the differences we observe. Other confounders, for example, the post-mortem interval between death and sample collection may also contribute (Ferreira et al., 2018). Moreover, as with any retrospective study it is possible that there were unknown, pre-existing differences between the two groups that contribute to the observed differences.

A further limitation of this study is that we cannot conclusively separate the influence of critical illness from the influence of critical care. Critical illness is itself associated with decreased tissue perfusion, hypoxia, and autonomic dysregulation–all of which may potentially impact clock function. While many of the subjects who died on a ventilator had traumatic events or acute medical emergencies (e.g., intracerebral hemorrhage) as a primary cause of death, these emergencies are often coupled with hemodynamic collapse and tissue hypoperfusion. Sepsis and secondary infection are also common. Nonetheless, we can use other data to help evaluate the hypothesis that critical care was itself a contributing factor. Other investigators (Cedernaes et al., 2018; Defour et al., 2020) have studied the influence of sleep loss and fasting–two unfortunate biproducts of current critical care protocols - on transcript expression in human adipose tissues. Notably, the genes identified as being influenced by fasting and sleep deprivation had minimal overlap (Jaccard coefficient = 0.017). Yet both gene sets were heavily enriched among the genes most significantly differentially expressed between the AD and IC groups. We further separated these gene sets into groups that were distinctly up (or down) regulated in the previous sleep deprivation and fasting experiments. These subgroups were again significantly enriched (Table 4). The strong overlap of fasting related genes with those modulated in the IC group is also consistent with our finding that clock function was generally most disrupted in metabolic and digestive tissues.

**TABLE 4:** Potential influence of sleep loss and fasting in IC group: Previous studies (Cedernaes et al., 2018; Defour et al., 2020) identified genes differentially expressed in human adipose tissue following sleep loss or fasting.

Enrichment of IC-effect genes for critical care gene sets	
Existing Gene Set	*p* values
Sleep Loss—bidirectionally regulated	0
Sleep Loss—up regulated	0
Sleep Loss—down regulated	0.03
Fasting—bidirectionally regulated	0
Fasting—up regulated	0.03
Fasting—down regulated	0

These gene sets have minimal overlap (Jaccard coefficient = 0.017). Enrichment analysis (fGSEA) is used to compare these genes sets with the influence of critical care. Genes were ranked by the significance (-log (FDR)) of the differential expression analysis comparing the AD and IC groups in adipose tissue. Enrichment analysis shows that IC affected genes are heavily enriched with genes modulated (both up or down regulated) by fasting or sleep loss.

Finally, we must emphasize that our analysis cannot demonstrate any prognostic difference related to circadian dysregulation. All subjects died prior to sample collection. A dedicated study would be required to show the impact of tissue specific clock dysfunction on mortality or other outcomes. However, our study does demonstrate that there is evidence of core clock dysfunction in the tissues of IC patients. Moreover, the transcriptomic changes we observe in the IC group mirror those observed following fasting and sleep deprivation. Taken together, these findings suggest that correcting circadian dysregulation has potential to improve critical care and that improving sleep and regularizing nutrition may advance that goal.

## Data Availability

Publicly available datasets were analyzed in this study. This data can be found here: https://www.gtexportal.org/home/datasets. The scripts used for data analysis, figure generation and the R files required for covariate corrected DCCD analysis are available at https://github.com/ranafi/ICU_clock_disruption.
