# Supplementary figures and images for: Multi-tissue transcriptional changes and core circadian clock disruption following intensive care
Source: Front Physiol. 2022 Aug 15;13:942704. doi: 10.3389/fphys.2022.942704 (PMC9420996; doi:10.3389/fphys.2022.942704)

(A)

Intensive Care (IC) and Acute Death (AD)  
Sample Groups

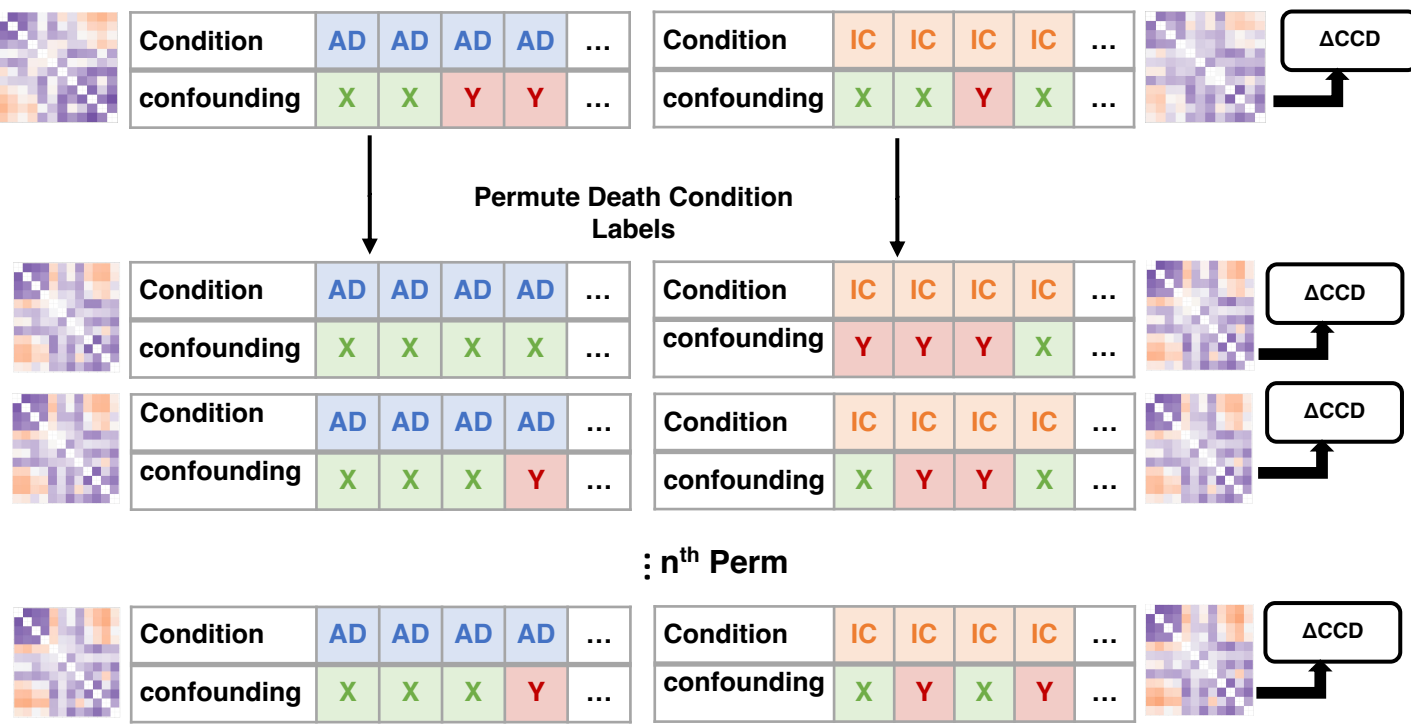

Supplement: Supplementary file 1 [file DataSheet2.PDF]
