# Supplementary material for: Multi-tissue transcriptional changes and core circadian clock disruption following intensive care
Source: Front Physiol. 2022 Aug 15;13:942704. doi: 10.3389/fphys.2022.942704 (PMC9420996; doi:10.3389/fphys.2022.942704)
Supplement: Supplementary file 4 [file DataSheet3.PDF]

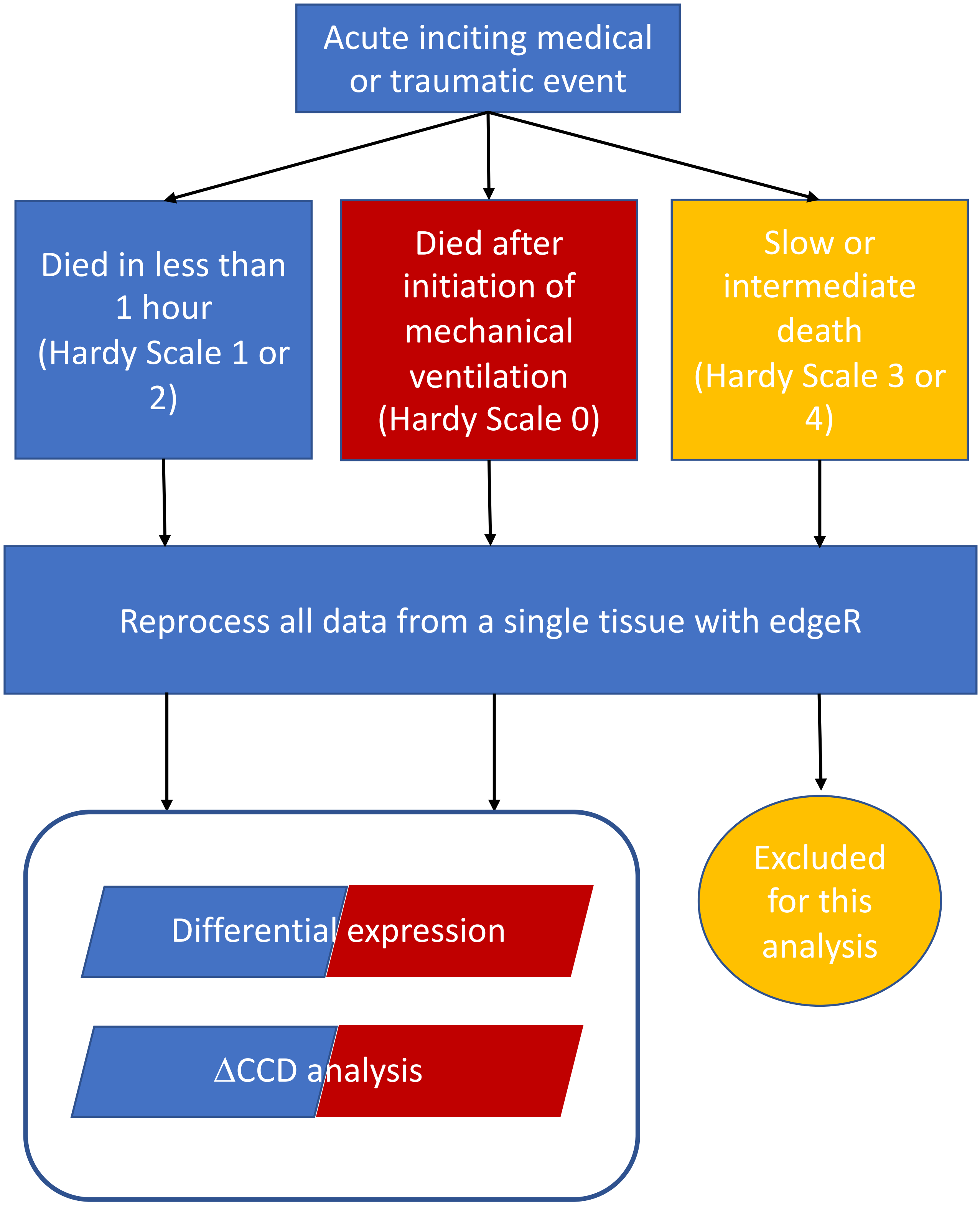

Acute inciting medical or traumatic event
Died in less than 1 hour (Hardy Scale 1 or 2)
Died after initiation of mechanical ventilation (Hardy Scale 0)
Slow or intermediate death (Hardy Scale 3 or 4)
Reprocess all data from a single tissue with edgeR
Differential expression
ΔCCD analysis
Excluded for this analysis
