# Supplementary material for: Multi-tissue transcriptional changes and core circadian clock disruption following intensive care
Source: Front Physiol. 2022 Aug 15;13:942704. doi: 10.3389/fphys.2022.942704 (PMC9420996; doi:10.3389/fphys.2022.942704)
Supplement: Supplementary file 5 [file DataSheet1.PDF]

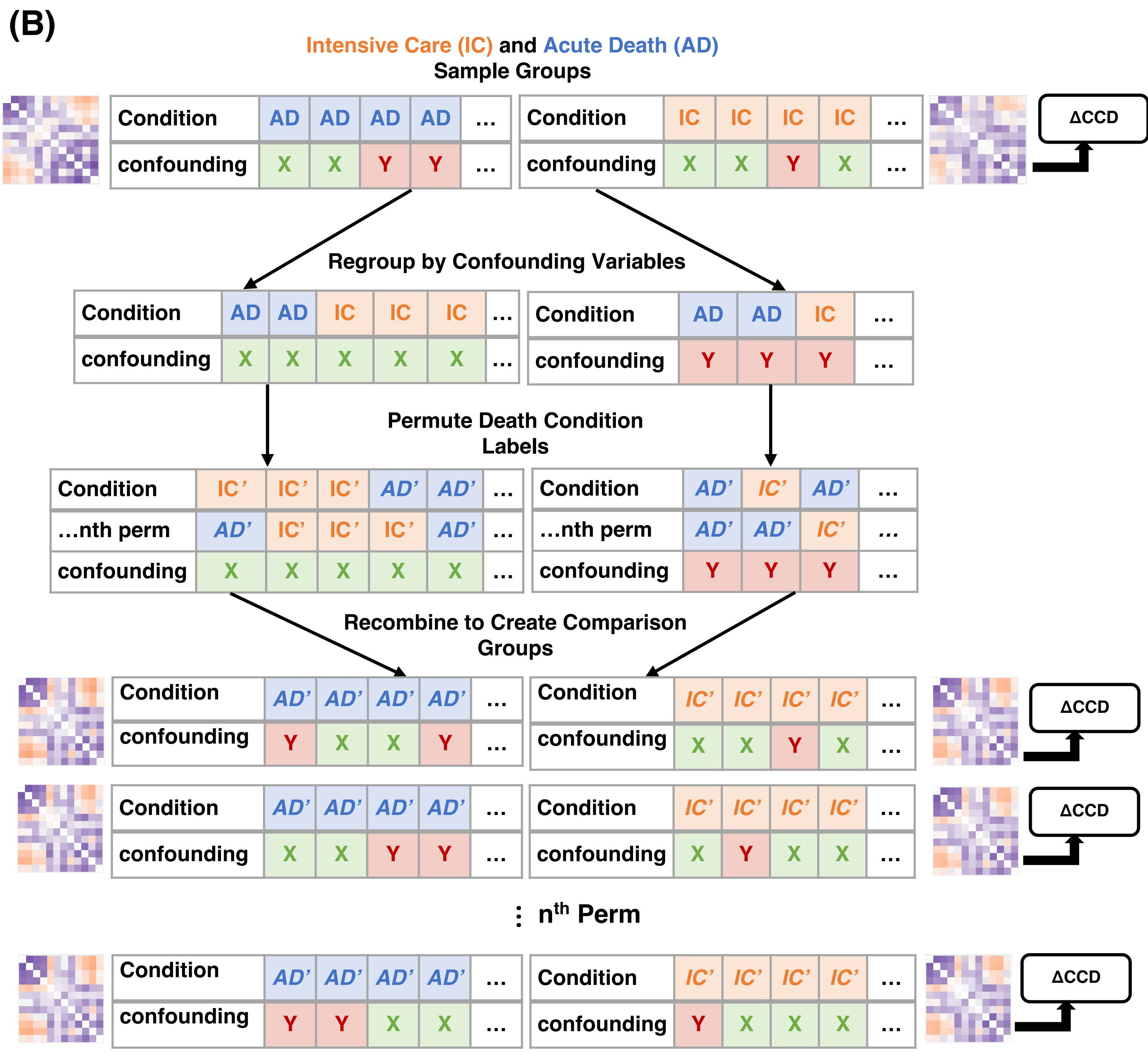
(B)
Intensive Care (IC) and Acute Death (AD)
Sample Groups
Condition AD AD AD AD ...
confounding X X Y Y ...
Condition IC IC IC IC ...
confounding X X Y X ...
ΔCCD
Regroup by Confounding Variables
Condition AD AD IC IC IC ...
confounding X X X X X ...
Condition AD AD IC ...
confounding Y Y Y ...
Permute Death Condition Labels
Condition IC' IC' IC' AD' AD' ...
...nth perm AD' IC' IC' IC' AD' ...
confounding X X X X X ...
Condition AD' IC' AD' ...
...nth perm AD' AD' IC' ...
confounding Y Y Y ...
Recombine to Create Comparison Groups
Condition AD' AD' AD' AD' ...
confounding Y X X Y ...
Condition IC' IC' IC' IC' ...
confounding X X Y X ...
ΔCCD
Condition AD' AD' AD' AD' ...
confounding X X Y Y ...
Condition IC' IC' IC' IC' ...
confounding X Y X X ...
ΔCCD
⋮ n^th Perm
Condition AD' AD' AD' AD' ...
confounding Y Y X X ...
Condition IC' IC' IC' IC' ...
confounding Y X X X ...
ΔCCD
